# Strong and weak tie homophily in adolescent friendship networks: An analysis of same-race and same-gender ties

**DOI:** 10.1017/nws.2022.24

**Published:** 2022-09-14

**Authors:** Cassie McMillan

**Affiliations:** Department of Sociology & Anthropology, School of Criminology & Criminal Justice, Northeastern University, Boston, MA, USA

**Keywords:** adolescent friendship, homophily, valued ERGMs, tie strength, race, gender

## Abstract

While we know that adolescents tend to befriend peers who share their race and gender, it is unclear whether patterns of homophily vary according to the strength, intimacy, or connectedness of these relationships. By applying valued exponential random graph models to a sample of 153 adolescent friendship networks, I test whether tendencies towards same-race and same-gender friendships differ for strong versus weak relational ties. In nondiverse, primarily white networks, weak ties are more likely to connect same-race peers, while racial homophily is not associated with the formation of stronger friendships. As racial diversity increases, however, strong ties become more likely to connect same-race peers, while weaker bonds are less apt to be defined by racial homophily. Gender homophily defines the patterns of all friendship ties, but these tendencies are more pronounced for weaker connections. My results highlight the empirical value of considering tie strength when examining social processes in adolescent networks.

Adolescent friendships are more likely to connect peers who have various characteristics in common than those who do not. Young people tend to befriend those who share their race and gender (Goodreau et al., 2009; Mehta & Strough, 2009), hold similar understandings of their national identity (Umaña-Taylor et al., 2020), and take the same academic classes (Frank et al., 2013). The tendency towards same-attribute friendships, or homophily (McPherson et al., 2001), carries important implications for various individual-level outcomes of interest, as well as broader social phenomena. On the one hand, networks that are heavily segregated often preclude minority adolescents from educational and socioeconomic opportunities, which can perpetuate broader patterns of social inequality (Mollica et al., 2003; Moody, 2001). On the other hand, friendships among homogenous peers can inspire participation in healthier behaviors (McMillan, 2019) and provide greater levels of social support (Ibarra, 1993), particularly for individuals from marginalized groups (Zhou et al., 2020). Despite this complex nature, there remain many unanswered questions about the homophily that defines adolescents’ social worlds.

Homophily is often conceptualized as a uniform phenomenon that defines all adolescent friendships as equal, regardless of each relationship’s strength, connectedness, or frequency of interaction. Most empirical work does not consider whether processes of homophily operate identically for strong, intimate adolescent friendships and those bonds defined by weaker connections (e.g., Goodreau et al., 2009; Moody, 2001). However, we know that strong and weak ties differ in terms of both their character and function. Strong ties are understood to be defined by high levels of emotional closeness (Marsden & Campbell, 1984), a sense of mutuality (Friedkin, 1990), and interaction across multiple foci (Wellman & Wortley, 1990). Weak ties, on the other hand, tend to be characterized by less frequent communication (Reagans, 2011) and maintained for involuntary purposes (Wellman & Wortley, 1990). Given these differences, strong ties are often characterized by greater levels of social support (Homans, 1950), while weaker ties are more likely to diffuse new information and encourage social change (Burt, 2004; Granovetter, 1973; Kreager & Haynie, 2011). Despite the varying benefits of strong and weak ties, it remains unknown whether homophily in adolescent friendship networks differs according to tie strength, and if these patterns vary across contexts. For instance, strong ties may be more likely to connect similar peers than their weaker counterparts on some individual-level characteristics, while weak ties may tend to connect similar peers on other traits of interest.

The current project begins to consider the relationship between tie strength and same-attribute connections by analyzing a large sample of over 150 adolescent friendship networks where participants were asked to differentiate between their strong versus weak friendship ties. I apply valued exponential random graph models (ERGMs), a novel statistical network method, to estimate whether homophily processes differ according to tie strength. Specifically, I test for variations in racial and gender homophily since these demographic attributes are particularly salient to young people’s daily lives (Moody, 2001; Rose & Rudolph, 2006). Then, I examine whether the association between homophily and tie strength varies according to the broader context of the networks in which individuals are embedded.

## Background

### Homophily in adolescent friendship across contexts

According to the principle of homophily, social contact is more likely to be observed among those who are similar than those who are different. There are several reasons why homophily characterizes a variety of social relationships, including adolescent friendships (Frank et al., 2013; Haas & Schaefer, 2014). First, individuals may hold inherent preferences to form ties with peers who are similar rather than those who are different (McPherson et al., 2001). Second, minority youth are often excluded from the social circles of majority group peers due to processes of discrimination and other structural barriers (McMillan, 2019; Tsai, 2006). This exclusion limits minority adolescents’ opportunities to form connections that cross social boundaries. Finally, social ties are more likely to connect those who are physically close and have frequent contact, yet geographic space and social structures tend to segregate individuals (Mouw & Entwisle, 2006). Adolescent tie formation, for example, tends to occur within schools that are organized by grade levels and systems of academic tracking, leading adolescents to spend more time with those of similar ages, socioeconomic backgrounds, and levels of ability.

Although adolescents tend to form relational ties with peers who share their same demographic traits and behaviors, the magnitude of this homophily can vary across school and community contexts. Blau’s (1977) macro-structural theory of intergroup relations emphasizes how various context-level phenomena can impact patterns of homophily in social interaction. As communities become more diverse on a characteristic of interest, for example, the probability that individuals from different groups will come into contact increases, and this may encourage the development of cross-group relational ties. Alternatively, when an individual has access to larger numbers of peers who identify with the same social groups as their own, they should report more homogenous connections than would be expected otherwise. These links between homophily and population composition have inspired researchers to consider how network context facilitates interaction among homogenous versus heterogenous pairs (e.g., McFarland et al., 2014; Moody, 2001; Smith et al., 2016).

Given their salience to adolescents’ daily lives, previous scholarship pays particular attention to how the social boundaries of race and gender shape the formation of youth friendships across various school and community contexts. There exists strong and consistent evidence that racial and ethnic homophily defines adolescent friendship networks (Goodreau et al., 2009; Moody, 2001), as well as patterns of romantic relationships (Kao et al., 2019) and even negative ties of bullying and aggression (Felmlee & Faris, 2016). A large part of the reason that we observe racial homophily is because the pools of potential friends that individuals can pick from tend to be defined by relatively high levels of racial homogeneity (McPherson et al., 2001). Many foci of social activity, such as schools, neighborhoods, and places of worship, are highly segregated by race. In these settings, individuals may form same-race ties because they have few opportunities to meet and interact with those of different racial backgrounds than their own. Yet even after taking a foci’s racial distribution into account, research finds that young people are more likely to form same-race friendships than would be expected by random chance (Goodreau et al., 2009; Moody, 2001; Shrum et al., 1988). Due to both personal preferences and exclusion, the social networks of adolescents tend to be segregated by race, even when opportunities for cross-race tie formation are present. In fact, network-level racial diversity and racial segregation tend to be *positively* correlated. In the US, school contexts characterized by higher levels of racial diversity tend to see more racial homophily in friendship patterns, though this tendency levels off and even begins to decrease in those networks that are the most diverse (Moody, 2001). Higher levels of diversity are also associated with increased racial/ethnic homophily in European contexts (Smith et al., 2016). Previous work finds that this association is linear for native-born, European adolescents, while the tendency for immigrant youths to seek out same-ethnicity friends peaks in classrooms defined by middling levels of diversity (Smith et al., 2016)

While the magnitude of gender homophily varies across the life course, previous work finds that gender is a prominent boundary in the social worlds of adolescents (Mehta & Strough, 2009; Rose & Rudolph, 2006). Part of the reason these patterns emerge is because social processes, like gender socialization (Chodorow, 1978) and gender performance (West & Zimmerman, 1987), lead girls and boys to enter friendships with varying expectations for these relationships. Gender differences in desires for intimacy, support, and involvement in shared activities may also lead young people to prefer same-gender friends (Rose & Rudolph, 2006). Unlike race, many foci of adolescent life contain relatively equal numbers of girls and boys. While context-level gender segregation tends to play a more modest role in explaining patterns of gender homophily, previous work finds that individuals who belong to networks defined by greater gender heterogeneity report more same-gender friendships (McFarland et al., 2014). These variations emerge because as individual characteristics, such as gender, become more universal within an environment, they tend to hold less importance for the formation of social relationships (Frank et al. 2008).

### Homophily and tie strength

Despite the attention given to homophily in the study of adolescent friendships, relatively little work considers whether these patterns are associated with tie strength. Instead, most empirical research tests whether homophily defines all friendship patterns in the same manner, regardless of each dyad’s emotional intimacy or the amount of time the pair spends together (e.g., Goodreau et al., 2009; Moody, 2001). Yet there is reason to believe that homophily operates differently according to the strength of relational bonds. Adolescent friendship networks may be characterized by *strong tie homophily*, or the tendency for highly intimate and close relational ties to cluster together peers who are similar, rather than those who are unalike. These networks could also be defined by *weak tie homophily*, or the tendency for bonds defined by lower levels of connectedness to link same-attribute actors (McMillan, 2022). While both strong and weak tie homophily are apt to define patterns of friendship for some attributes of interest, friendship networks may only be characterized by strong or weak tie homophily for other traits. This is because strong and weak relationships serve different social functions and develop within varying structural contexts.

Theoretical work argues that strong ties tend to be exhibit greater levels of social support (Homans, 1950), while weak ties are more likely to circulate new information and inspire social change (Burt, 2004; Granovetter, 1973). Given that individuals rely on their close connections for instrumental and emotional resources (Wellman & Wortley, 1990), adolescents may explicitly prefer cultivating emotionally close friendship ties with peers who have similar lived experiences as their own. Although cross-race friendships hold promise for reducing interpersonal prejudice (Allport, 1954; Pettigrew & Tropp, 2006), relationships with same-race peers can support emotional well-being and nurture racial identity formation, particularly among ethnic minority youth (Killen et al., 2021; Kornienko & Rivas-Drake, 2021). Strong, same-race ties may help facilitate the advantages of these homogenous connections, while this shared background is apt to be less pertinent, or even detrimental, to the formation and maintenance of weaker connections.

Alternatively, weak ties are hypothesized to carry the most benefits when they link pairs who would otherwise be socially distant (Granovetter, 1973). We know that people form relationships across the different foci that define their daily routines (Feld, 1981), yet the structures that encourage and restrict adolescents’ opportunities to meet one another are defined by varying levels of segregation. Schools that adhere to systems of academic tracking often schedule classes that are segregated by race (Killen et al., 2021), for example, while extracurricular activities tend to be more racially integrated (Quiroz et al., 1996). In environments like these, strong cross-race ties are apt to develop between peers who played on the same sports team across multiple years, while weaker same-race ties may connect peers who took a class together during a single semester.

Previous empirical work finds some evidence that tie strength is associated with tendencies towards, or against, racial and gender homophily. For example, the developmental literature suggests that friendship bonds connecting youth from different racial backgrounds tend to be weaker than those that link same-race pairs (Echols & Graham, 2013; Kornienko & Rivas-Drake, 2021). Cross-race friendships are often characterized by lower levels emotional closeness (Aboud et al., 2010) and more asymmetry in nomination patterns (Vaquera & Kao, 2008). Through the analysis of various types of adolescent networks, Windzio & Bicer (2013) find that racial and ethnic segregation is more pronounced for “low-cost” relationships, such as friendship nominations, than those that are “high-cost,” like meeting a peer’s parents. They argue that ethnic boundaries make it difficult for actors to develop and maintain high-cost ties with cross-ethnic peers because establishing these relationships can be challenging and defined by more uncertainties.

Another line of recent work analyzes novel data sources to consider the relationship between gender homophily and frequency of interaction. Through the collection of data from wearable sensors, Stehlé et al. (2013) construct young children’s social networks in which ties are weighted by each dyad’s frequency of interaction. They find that girls and boys are more likely to report strong ties with same-gender peers, but that the tendency towards weak tie gender homophily is less pronounced. On the other hand, Mollgaard et al. (2016) use data on text messaging patterns among a population of college students to assess the relationship between tie strength and gender homophily. Ties characterized by greater volumes of text messages connect cross-gender peers more than same-gender peers – a finding that the authors attribute to heterosexual romantic relationships.

### Current study

Despite this previous work, there remain several unanswered questions about the association between tie strength and homophily in adolescent friendship. In the current project, I explore this relationship by asking whether tendencies towards homophily differ for strong versus weak adolescent friendship ties by considering two demographic characteristics of interest – race and gender. Both race and gender represent important boundaries in the social worlds of adolescents, but their impact on the formation of strong versus weak homogenous ties should vary in distinct ways. Extending prior literature on the connection between racial homophily and tie strength (Echols & Graham, 2013; Windzio & Bicer, 2013), I expect that both strong and weak ties will be more likely to connect same-race peers, but that this tendency will be even greater for ties defined by high levels of strength (Hypothesis 1). Next, I anticipate that gender homophily will also define young people’s social ties but that patterns will differ for strong versus weak connections. Given that heterosexual romantic relationships become increasingly common in adolescence (Mollgaard et al., 2016), I hypothesize that patterns of strong tie gender homophily will be less pronounced than patterns of weak tie gender homophily (Hypothesis 2).

Next, I consider whether strong and weak tie homophily vary according to contextual factors since the general tendencies towards same-race and same-gender adolescent friendships are not consistent across school environments. First, I test whether patterns of strong versus weak tie racial homophily vary according to a network’s racial distribution since previous work finds that friendships are more likely to connect same-race adolescents in racially heterogeneous environments (Goodreau et al., 2009; Moody, 2001). I hypothesize that this trend is driven by patterns of strong ties and suspect that the tendency towards strong tie racial homophily and network-level racial heterogeneity will be positively correlated, while this association will be less pronounced for weaker ties (Hypothesis 3). Finally, I consider whether patterns of strong versus weak tie gender homophily differ according to a network’s gender composition since previous work finds that gender heterogeneity is associated with network structure (McFarland et al., 2014). I anticipate that weak tie gender homophily will reach its highest levels in schools with unequal gender distributions, while these patterns will be less pronounced for strong tie gender homophily because of heterosexual dating patterns (Hypothesis 4).

## Data and methods

### Sample

I utilized data on over 14,000 students who participated in the Promoting School-Community Partnerships to Enhance Resilience (PROSPER) study. All respondents attended school in one of 28 public-school districts during their sixth through twelfth grade years. Participating districts were located in rural communities or small cities in Pennsylvania or Iowa and half were randomly selected to receive a substance abuse prevention campaign that included community- and school-based components. The study collected eight waves of panel data for two cohorts of students. One cohort began sixth grade in 2002 and the other started in 2003. Self-administered surveys were distributed at each school during the fall and spring of students’ sixth grade years and during the spring of students’ seventh through twelfth grade years. Both response and retention rates remained relatively high throughout the survey. Across all waves, 86%–90% of eligible students participated, and of the students who were present at the first wave of the study, each participated in an average of 5.90 waves.

In the current project, I specifically focused on survey data collected at three waves of the study: the fall semester of students’ sixth grade year (Wave 1) and the spring semesters of students’ eighth grade (Wave 4) and eleventh grade years (Wave 7). These three waves represent distinct developmental periods in which homophily processes may operate differently (Killen et al., 2021; Mehta & Strough, 2009). For these analyses, it was necessary to omit students who attended certain schools due to inconsistencies with data collection (e.g., one community was affected by a fire). After these omissions, my sample included an average of 8,530 students per wave and all students hailed from one of 51 school networks.

### Survey items

#### Friendship nominations

Friendship data were collected at each wave of the survey by asking students, “who are your best and closest friends in your grade?” Respondents could nominate up to two “best friends” and five “other friends.” Following previous work (McMillan, 2022; Shrum et al., 1988), I defined best friend nominations as *strong ties* and other friend nominations as *weak ties*.^1^ While the distinction between best and other friends represents a subjective categorization of tie strength, respondents were also asked how often they “spend time just ‘hanging out’” with each nominated friend outside of school without adult supervision (responses ranged from 1 = *never* to 5 = *almost every day*). Results of a two-tailed *t*-test (*p* < 0.001) demonstrated that respondents spent significantly more time with their best friends (mean = 2.45, SD = 1.36) than their other friends (mean = 1.82, SD = 1.37), providing some evidence that best friendship nominations were characterized by higher objective levels of tie strength. As an additional robustness check, I considered whether the different nomination caps for best versus other friends shaped my findings for a subsample of networks and found no evidence that this was the case (see Supplemental Materials, Part B). For both friendship categories, I included only within-school and within-grade nominations as this allowed me to link each respondent to the individual-level data of all peers they nominated.

#### Sociodemographic variables

To test my research questions, I constructed a binary variable for individual gender where 1 = *boy* and a variable for race that used two survey items asked at each wave of the survey. First, respondents were asked if they identified as Hispanic or Latino. Then, they were asked to select a racial category that best described them from “Native American/American Indian,” “Black/African American,” “Asian,” “White,” “more than one of those listed,” and “other.” Given the low amount of racial diversity in the PROSPER sample, it was necessary to recode race into four categories: (1) *any race Hispanic/Latino*, (2) *Black and non-Hispanic/Latino*, (3) *White and non-Hispanic/Latino*, and (4) *other racial group and non-Hispanic/Latino*.

I also constructed an individual-level measure of socioeconomic status (SES) because prior work finds that people tend to befriend those from similar socioeconomic backgrounds as their own (McPherson et al., 2001). Since the in-school PROSPER survey did not ask respondents to report their household income or parents’ highest education level, previous work uses a survey item that asked students whether they received free or reduced school lunch as a proxy for SES (Copeland et al., 2017; Osgood et al., 2014). I coded the SES proxy as a binary variable where 1 = *receives free or reduced lunch*. Since missing data was relatively low (less than 5% of cases for all variables of interest), I used list-wise deletion to account for missingness in my sample (following Allison, 2001).

### Plan of analysis: valued ERGMs

To address my research questions, I applied valued ERGMs to disentangle how different types of strong and weak homophily shape adolescents’ friendship patterns (Krivitsky, 2012). These are state-of-the-art, multivariate models from the ERGM family that employ a statistical analysis to compare the dyadic, or pairwise, patterns observed in an actual network to what would be expected by random chance. By making this comparison, valued ERGMs can determine whether the processes observed in an actual network are statistically significantly different from what would be expected to occur randomly, after controlling for all other included variables.

Traditional ERGMs, or p* models, can only analyze binary network data, or data where we know only whether a tie is present or absent (Hunter, 2007; Robins et al., 2007; Wasserman & Pattison, 1996). However, recent scholarship has generalized the ERGM to evaluate networks where dyads are weighted with count values (Krivitsky, 2012; Cranmer et al., 2020). The ERGM for weighted networks follows a similar statistical process as the binary ERGM, which allows it to maintain much of the traditional, binary ERGM’s flexibility. After a valued ERGM achieves convergence and adequate goodness-of-fit statistics, coefficient estimates can be interpreted in a manner similar to how one would consider coefficients from a logistic regression. However, unlike a traditional logistic regression, results are interpreted at the level of the dyad rather than the individual.

In a valued ERGM, **Y** is defined as an *n* × *n* matrix (where *n* equals the number of actors) such that the (*i*, *j*) entry of this matrix is an integer that indicates each relational tie’s associated weight if there exists a connection between actors *i* and *j*. If no tie exists, then (*i*, *j*) is set to 0. The valued ERGM specifies the probability that network **Y** will occur given a set of individuals:

$$P(Y=y|X)=\frac{h(y)\exp\left[\theta^{T}g(y)\right]}{k_{h}(\theta )},y\in y.$$

Here, **X** represents a matrix of covariates and *θ* is a vector of all network coefficients that are hypothesized to relate to the probability of the observed network’s structure. A vector of network statistics, *g*(*y*), is calculated using the observed adjacency matrix, and *k*_*h*_(*θ*) is a normalizing factor that ensures that the result is a legitimate probability distribution.

There are two key differences between traditional binary ERGMs and weighted ERGMs. First, the weighted ERGM compares the edge values to the observed network to a support (*y*), or a sample space of all possible values that each dyad can take. This sample space is also considered in the normalizing factor of the model’s equation (*k*_*h*_(*θ*)). Second, the weighted ERGM requires the researcher to specify a reference distribution (e.g., binomial or Poisson distribution) to model the distribution of tie weights. This reference distribution shapes the value of the *h* function in the numerator of the valued ERGM equation. My reference distribution in the current project was a binomial distribution with the following tie weight categories: 0 = *no friendship tie*, 1 = *other friends*, and 2 = *best friends*.

#### Valued ERGM parameters

I included two types of parameters in each valued ERGM: the first measured structural processes that are endogenous to the network and the second related to homophily on specified individual-level attributes. Four of the included parameters accounted for structural tendencies of interest. First, I included the *sum* parameter which added together the values assigned to all edges in the network. The sum parameter can be understood to play a similar role as an intercept in a regression. Second, the *nonzero* parameter measured the likelihood that a friendship tie of a non-zero value existed between any two actors in the network. The nonzero parameter helped account for the fact that friendship networks tend to be relatively sparse, or zero-inflated. I also included a *mutual* parameter to measure tendencies towards reciprocated ties (e.g., adolescent *a* nominated adolescent *b* as a friend and *b* nominated *a* as a friend), as well as a *transitivity* parameter to account for patterns of triadic closure (e.g., if *a* was friends with *b* and *b* was friends with *c*, then *a* and *c* were also friends). I constructed both the mutual and transitivity parameters following the criteria suggested by Krivitsky & Butts (2018).

I included two types of homophily parameters for several individual-level attributes across the valued ERGMs that I estimated. I refer to the first variant as *general homophily*, which assumed that processes of homophily were consistent, regardless of tie strength. The general homophily parameters did not differentiate between best and other friendships. In other models, I included a second type of homophily parameters that allowed the process to vary for ties with stronger versus weaker weights (following McMillan, 2022). In this case, I included two parameters simultaneously: one for *strong tie homophily* and another for *weak tie homophily*. The strong tie homophily parameter measured tendencies towards maintaining social ties to same-attribute peers at or above a specified weight value. The weak tie homophily parameter measured the tendency for social ties to form between same-attribute peers that are below a specified weight value. I selected cutoff values such that the strong tie homophily parameter only considered tendencies towards best friendship (weight ≥ 2), while the weak tie homophily parameter only evaluated patterns of other, non-best friendships (weight < 2). Across the ERGMs I estimated, I included a variety of general homophily terms for shared race, gender, and SES. To test whether tendencies towards forming same-race and same-gender friendships vary across strong versus weak ties, I estimated additional ERGMs that included strong and weak tie homophily coefficients for either race or gender.

#### Meta-analysis

Generally, valued ERGMs can only analyze a single network at a time without experiencing convergence issues. As a result, I estimated models on each of the 153 networks separately until each valued ERGM reached convergence, produced satisfactory fit statistics, and exhibited low risk of multicollinearity (see Supplemental Materials, Part C). To summarize the findings across my sample of networks, I aggregated results from each model by employing a two-level random effects meta-analysis. This averaging process accounted for the different levels of precision across all models by giving greater weight to those with more precise estimates. One ERGM that estimated strong and weak tie racial homophily and three that considered both variants of gender homophily were unable to converge and were excluded from the meta-analysis.

### Plan of analysis: multilevel regression models

To compare the prevalence of homophily across different contexts, I next estimated a series of multilevel models (MLMs). In my sample, there were three wave-specific networks nested within each of the 51 school-based cohorts. I accounted for the nested structure of the data by constructing longitudinal MLMs with two levels where the first represents the wave-specific network (*n* = 153) and the second represents the school-based cohort (*n* = 51). For both race and gender, I estimated three multilevel logistic regression models where the dependent variables were the coefficient values for one of the ERGM homophily parameters. In the first set of MLMs, the dependent variable was the coefficient for the general homophily term included in the first set of ERGMs that did not distinguish between patterns of homophily for strong versus weak ties. The dependent variable in the second model was the coefficient values for the strong tie homophily parameter, and in the third model, it was the coefficient values for the weak tie homophily parameter.

To illustrate my multilevel modeling strategy, I outline my multilevel regression model for the general homophily coefficient (*Y*_*ij*_) below:

$$Y_{ij}=\gamma_{00}+\sum \gamma_{y0}W_{ij}+\sum \gamma_{0x}S_{j}.$$

This equation represents my combined model where *γ*_00_ is the intercept at the level of the school-based cohort. I included several variables at the level of the wave-specific network (*W*_*ij*_) and school-based cohort (*S*_*j*_). Note that the MLMs predicting coefficient values for high- and low-value homophily included similar components as those presented above.

To evaluate my third and fourth hypotheses regarding context-level heterogeneity, I included indicators of the racial and gender composition at the level of the wave-specific network. The network-level measure of *racial composition* was equal to the percentage of network actors who identified as racial/ethnic minorities, or those individuals who indicated they were Hispanic/Latino, Black, or members of another nonwhite racial group. My measure of *gender composition* considered the percent of network actors who identified as girls. Both measures were constructed using the respondent-level variables described previously.

All MLMs contained various controls at the level of the wave-specific network. First, I included a set of dummy variables to account for the wave of the study, or students’ grade level when surveys were administered (sixth, ninth, or eleventh grade). Since students who attended schools with greater numbers of peers have more options for potential friends, I constructed a count of the number of students enrolled in each grade cohort. Finally, I included controls for friendship homophily on additional individual characteristics that are known to shape friendship patterns (McPherson et al., 2001). In all models, I incorporated a measure of general homophily on SES. In MLMs where the dependent variable was racial homophily, I included a control for general homophily on gender. I included a control for general homophily on race in MLMs where the dependent variable of interest was gender homophily. All homophily controls were constructed using parameter estimates from my first set of valued ERGMs.

At the level of the school-based cohort, I included several controls that did not vary across the wave-specific networks. First, I constructed binary variables to indicate the state where the school was located (1 = *Pennsylvania*) and whether the school participated in the substance abuse prevention campaign (1 = *selected to participate*). Next, I included a control for the percent of each school district that was rural since the broader context of the community in which a school district is located can shape students’ opportunities to meet and form friendships, as well as school bussing patterns (Moody, 2001). Finally, I controlled for the average amount of money spent per student (in thousands of dollars) to account for the economic resources of the school district. I included this control because access to financial resources is likely to shape administrators’ efforts to facilitate the formation of heterogenous friendships (McFarland et al., 2014).

## Results

While the majority of adolescents in my sample identified as white, 6.49% identified as Hispanic or Latino, 3.22% identified as Black, and 5.70% identified as another race (see Table 1). Additionally, the sample included roughly equal numbers of girls and boys. Despite these general trends, it is important to note that substantial variations existed across the 153 networks. For instance, the percent of racial minority students varied from 1.19% in the least racially diverse network of the sample but exceeded 45% in the most diverse community. There were also variations in the gender distributions across my sample of networks. The percent of girls varied from 34.52% to 63.16%, with the average network reporting a student body that was 51.13% female.

### ERGM meta-analyses

Complementing previous work, I found strong evidence for homophily on race and gender when tie strength was not taken into account (see Table 2, Model 1). After controlling for a variety of structural and dyadic phenomena, adolescents were roughly 1.14 times more likely to nominate a same-race peer as either a best friend or non-best friend when compared to a cross-race peer (*b* = 0.128, *p* < 0.001). At the same time, same-gender dyads were 3.75 times more likely to occur than cross-gender dyads (*b* = 1.322, *p* < 0.001).

Control variables also shaped patterns of friendship in the expected directions. The coefficients for the sum and nonzero parameters were negative and significant (*b* = −5.650, *p* < 0.001 and *b* = −1.057, *p* < 0.001, respectively), suggesting that the weighted friendship networks were relatively sparse. Respondents also tended to reciprocate friendship nominations and send ties that result in triadic closure, as demonstrated by the positive and significant coefficients for mutuality (*b* = 2.566, *p* < 0.001) and transitivity (*b* = 0.847, *p* < 0.001). Finally, I uncovered evidence for homophily on SES (*b* = 0.164, *p* < 0.001). Adolescents were 1.18 times more likely to befriend peers who shared their free/reduced lunch status than those who did not, net of all included parameters.

When strong and weak tie homophily were considered separately, however, I found evidence that the social process can vary according to tie strength. Contrary to my first hypothesis, I uncovered evidence for weak tie homophily on race, but no support for strong tie racial homophily (Table 2, Model 2). An adolescent was 1.24 times more likely to nominate a same-race peer as a non-best friend than a cross-race peer, net of all controls (*b* = 0.219, *p* < 0.001). At the same time, respondents were not statistically significantly more likely to send best friend nominations to same- versus cross-race peers (*b* = 0.012, *p* = 0.815). With regards to gender homophily, the adolescents in my sample were more likely to send strong and weak ties to same-gender peers, but these processes varied in their magnitude (Table 2, Model 3). Respondents were 2.62 times more likely to nominate a same- versus a cross-gender best friend (*b* = 0.963, *p* < 0.001) and 4.06 times more likely to nominate a same- versus a cross-gender other friend (*b* = 1.400, *p* < 0.001), which gives support to my second hypothesis.

In supplemental analyses, I also considered whether the magnitude of general, strong tie, and weak tie homophily varied as a function of respondent age (see Supplemental Materials, Part B). For gender, the tendency towards all three variants of homophily significantly declined for respondents in older age groups. There was some evidence that the magnitude of weak tie racial homophily increased for older adolescents (*Weak tie homophily* × *11th grade*: *b* = 0.102, *p* < 0.05); however, no other interactions with the racial homophily terms were statistically significant.

### Variation across networks: multi-level regression models

#### Racial homophily

To examine the factors associated with network-level variation in strong and weak tie homophily, I next estimated several multi-level regression models. The first set of models tested whether the racial distribution of each network was associated with the three different types of racial homophily, while controlling for various network- and community-level factors. Following previous work (Moody, 2001), I included third-order polynomials for the measure of racial distribution in these models.^2^

I did not find a significant association between network racial composition and the general racial homophily coefficient (see Table 3, Model 1). When strong and weak tie homophily on race were considered separately, however, I uncovered evidence for a significant, nonlinear relationship between racial composition and tendencies towards same-race weak ties (see Table 3, Model 2). The third-order polynomials for racial composition suggested that among schools with predominately white student bodies, increasing the level of racial diversity led to a decline in weak tie racial homophily (*b* = −0.100, *p* < 0.01). However, this decline only persisted until a certain point, at which higher levels of school diversity led to increased racial homophily among weak ties (*b* = 0.005, *p* < 0.01), though the impact of this positive association eventually leveled off (*b* = −0.00006, *p* < 0.05). It is important to note that the polynomials for racial composition did not reach statistical significance in the model predicting the strong tie homophily (Table 3, Model 3). However, all three polynomials for racial composition in the model predicting strong tie racial homophily (Model 3) were significantly different from those in the model predicting weak tie homophily (Model 2), according to a Chi-squared test (*p* < 0.05).

**Figure 1.**
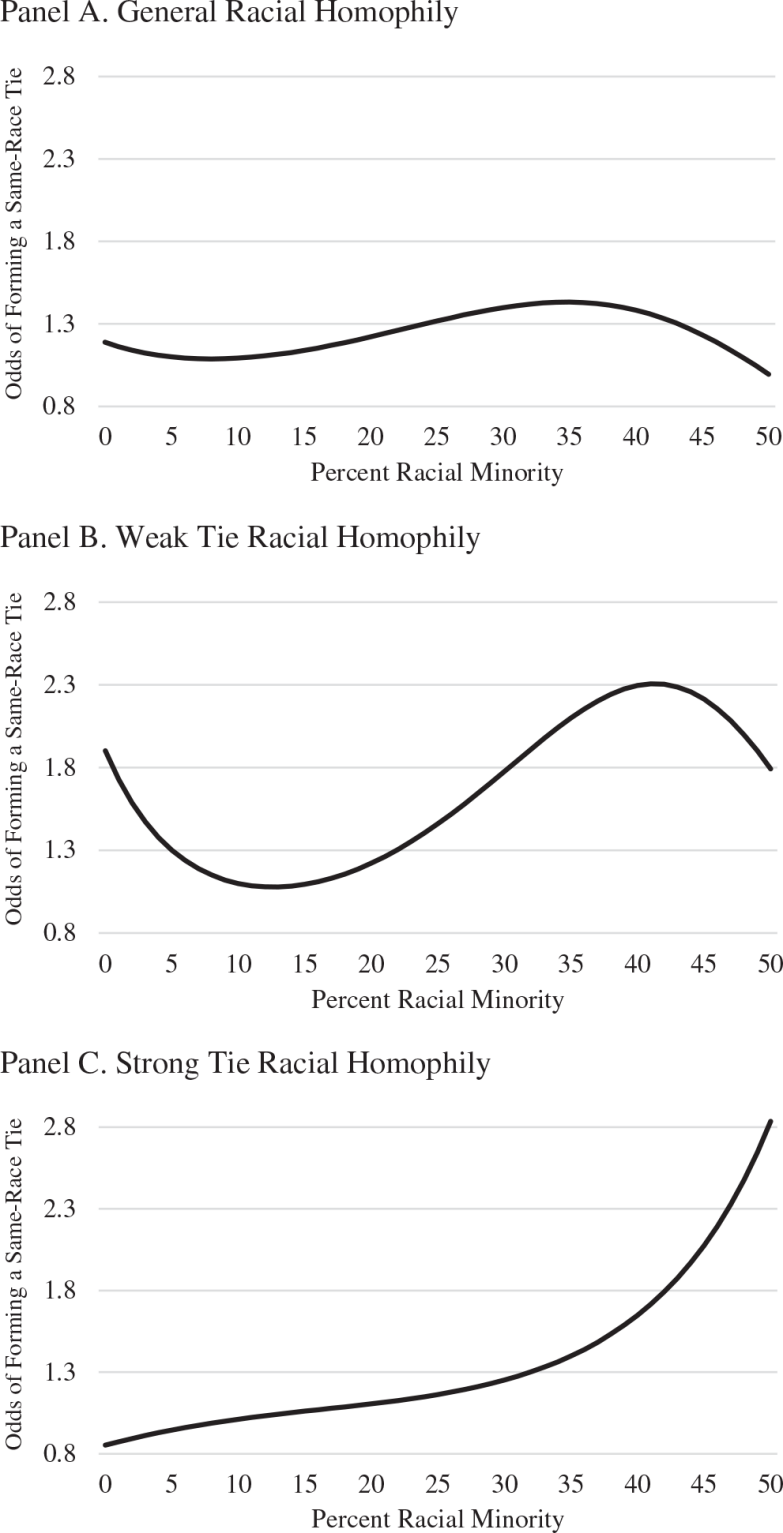
Predicted odds of forming a same-race tie for all, best, and other friendships. Expected odds for eighth graders are presented. Control variables are set to their mean value.

Given the challenges of interpreting third-order polynomials, I present a graph to demonstrate the relationship between the network-level distribution of race and an individual’s odds of forming certain ties with same-race peers (see Figure 1). Here, I plot the odds of reporting a same-race tie for the eighth graders in my sample (i.e., the exponentiated values of the *b* coefficients), while all other control variables were set to their mean value. Findings suggest that general and weak tie racial homophily were at their lowest levels in nondiverse settings but intensified as the proportion of minority students increased. After a certain tipping point (roughly 40% racial minority students), however, this tendency towards forming same-race ties began to decrease (see Panels A and B). The formation of strong ties, however, did not experience this same tipping point. Instead, as schools became more diverse, the tendency to report same-race best friendship increased steadily (see Panel C). Taken together, these findings lend partial support to my third hypothesis.

#### Gender homophily

Next, I considered a set of models that tested whether the gender composition of each network was associated with general, strong tie, and weak tie homophily on gender. For these models, I included a set of second-order polynomials to test for the association between the proportion of female actors in a network and the different variants of gender homophily in my sample of networks.^3^ Contrary to my fourth hypothesis, I found no evidence that a network’s gender composition significantly shaped patterns of general, strong tie, or weak tie gender homophily (see Table 3). The second-order polynomials were similar in their size and direction across all three models, as confirmed by a Chi-squared test.

## Discussion

Although most previous work on homophily in adolescent networks overlooks variations in the intimacy or connectedness of social ties, the current project argues that we can gain additional insight into young people’s social worlds by considering strong and weak tie homophily separately. Across a sample of 153 adolescent friendship networks, I found that there were key differences in strong and weak tie homophily on race. When data from all schools were aggregated, weak ties were more likely to connect same-race peers than cross-race peers, but there was no significant tendency towards racial homophily on strong ties. These associations varied according to the racial composition of one’s school, however. As the proportion of racial minority students increased, one’s odds of naming a same-race peer as a best friend sharply increased, while their odds of naming a same-race peer as a non-best friend slightly declined. Adolescents’ strong and weak ties were both more likely to connect them to same-gender peers than cross-gender peers, but the magnitude of weak tie gender homophily was greater than that for strong ties. The current study suggests that homophily can differ for strong versus weak ties, but that these patterns vary according to the demographic trait of interest and certain context-level factors.

On average, the adolescents in my sample were significantly more likely to form same-race weak ties, but no such tendency existed for strong ties. This pattern likely results from the fact that individuals form social relationships across a variety of different foci (Feld, 1981), some of which are more or less racially integrated than others. It might be the case that those foci that encourage the formation of long-lasting, emotionally close friendships are more racially integrated, such as sports teams or school clubs, than those that inspire the formation of weaker bonds, such as academic classrooms. Despite these general trends, it is important to note that the association between tie strength and racial homophily varied across the networks in my sample, particularly in relation to each network’s racial distribution. In fact, the insignificant tendency towards strong tie racial homophily in the aggregated results was driven primarily by processes occurring in predominately white schools. Within schools where most of the student body (i.e., over 90%) identified as white, measures of both strong and weak tie racial homophily were relatively low, although still statistically significant. These findings are not surprising since the salience of race and ethnicity tends to be minimal for white actors in predominantly white populations (Lewis, 2001). When racial boundaries are less prominent, other social divisions, such as those by socioeconomic status or age, are apt to play a more influential role in segregating patterns of social interaction (Frank et al., 2008; Moody, 2001).

As schools became more racially diverse, the odds of forming same-race best friendships also increased. This finding suggests that there are sizable tendencies towards strong tie racial homophily in schools where high proportions of the student body identify as racial minorities. When solely weak ties were considered, I observed an initial increase in weak tie racial homophily, but after a certain tipping point was reached (i.e., roughly 40% of the student body identifies as a racial minority) the odds of forming same-race other friendships began to decline. These findings align with previous work that considers the relationship between general racial homophily and the racial composition of networks (e.g., Moody, 2001; McFarland et al., 2014; Smith et al., 2016). General racial homophily defines adolescent friendship networks almost universally (Goodreau et al., 2009). However, the magnitude of this process experiences a paradoxical increase as school settings become more racially diverse, although this eventually levels off (Moody, 2001). By considering strong and weak ties separately, I find evidence that the link between network context and cross-race friendships further varies according to the strength of these relationships.

Future research should consider why racially heterogeneous networks report high levels of strong tie racial homophily, but more modest levels of weak tie racial homophily. Perhaps racial minority students who attend diverse schools report greater numbers of same-race strong ties because they have more opportunities to interact with peers who share their background than racial minority youth in predominately white schools (Moody, 2001). This shared racial background may be crucial for facilitating the high levels of social and emotional support that define strong ties (Homans, 1950; Ibarra, 1993). Weak ties, on the other hand, are applauded for their ability to bridge disparate groups and inspire the spread of novel information (Granovetter, 1973; Kreager & Haynie, 2011). Cross-race weak ties could be beneficial for adolescents since these connections have the potential to introduce the involved actors to new opportunities and ideas. At the same time, when racial and ethnic boundaries are salient, as is the case in racially heterogenous middle and high schools, the costs of developing intimate cross-race connections are higher and their maintenance is characterized by more uncertainty (Windzio & Bicer, 2013). In these contexts, social relationships between same-race peers are apt to be defined by greater levels strength and connectedness than bonds between cross-race peers.

Interestingly, I found fewer differences between strong and weak tie homophily on gender in my sample of adolescent friendship networks. Adolescents were more likely to form best and other friendships with same-gender peers, but these tendencies varied in their magnitude. The adolescents in my sample had higher odds of reporting same-gender non-best friendships than same-gender best friendships, and this finding was largely driven by the network patterns of older adolescents. These findings complement the developmental patterns documented in previous research (Furman & Rose, 2015; Poulin & Pedersen, 2007). Since adolescence is a period in the life course when heterosexual dating becomes increasingly common, for example, the higher odds of reporting different-gender strong ties could be due to respondents nominating their significant others as best friends (Mollgaard et al., 2016). Furthermore, I did not uncover a significant relationship between a network’s gender composition and the tendency towards forming friendships characterized by general, strong tie, or weak tie gender homophily. Instead, the link between tie strength and same-gender friendship was largely similar across various contexts. When interpreting this finding, it is important to note that most networks in my sample were characterized by relatively equal gender distributions. The association between tie strength and gender homophily may be more pronounced in networks where the distribution is highly skewed.

The current project demonstrates the utility of differentiating between strong and weak tie homophily when studying patterns of adolescent friendships. Making this distinction is apt to provide new insight into the broader implications of adolescent friendship patterns. For instance, some previous work finds that homophily is associated with positive, pro-social outcomes, such as better health (McMillan, 2019; Zhou et al., 2020) and receiving more social support (Ibarra, 1993). Other research highlights the negative consequences of homophily, including its ability to exclude minority groups from opportunities for socioeconomic advancement and implications for the development of prejudiced attitudes (Kornienko & Rivas-Drake, 2021; Mollica et al., 2003). Policymakers and school professionals should be aware of the ways that same-race and same-gender friendships can promote and challenge various inequalities. Findings from the present study confirm that simply introducing minority members to majority-dominated contexts is not sufficient to cultivate the benefits of diversity (Yip et al., 2019). Instead, school districts need to invest in developing academic courses and extracurricular activities that encourage the development of social ties that cut across social boundaries. Even if these bonds are relatively weak in their strength, they are likely to connect young people from minority groups to crucial resources and opportunities.

Although the current study has several strengths, it also has limitations. First, my sample of adolescent friendship networks was not nationally representative. Instead, all networks were collected from small cities and rural areas, many of which were characterized by predominately white populations. Future work should apply the analytical strategies presented here to more diverse samples of adolescent friendship networks. By analyzing samples with more racial heterogeneity, for example, we can gain insight into whether the magnitude of strong and weak tie homophily varies for pairs of youth who share racial majority versus racial minority backgrounds. Second, future work should analyze processes of strong and weak tie homophily by using datasets with more details on tie strength. While the adolescents in my sample reported spending significantly more time with their best friends outside of school, we know little about the relative closeness of “best” versus “other” friendship nominations, or whether this difference remained constant across participants. Thus, the analytical strategies discussed here could be used to analyze datasets with more detailed measures of tie strength, such as rankings of emotional intimacy. Third, students were permitted to nominate no more than two best friends and five other friends. This nomination cap may have prevented a small minority of participants from nominating all their relational ties, and as a result, the results reported herein may underestimate tendencies towards gender and racial homophily.^4^ Finally, the current study was only able to consider patterns of homophily among within-school and within-grade friendship ties. Thus, the conclusions reported here cannot be generalized to adolescent connections that form outside of one’s school-grade cohort.

Despite these limitations, the current project represents one of the first studies to disentangle processes of strong and weak tie homophily in a large sample of adolescent friendship networks. By considering both racial and gender homophily, I analyzed whether processes of strong and weak tie homophily operated differently for the two attributes of interest, as well as how these processes varied across a large sample of networks. For example, I found that tendencies towards same-race best friendships were particularly strong in the most racially diverse schools in my sample, while tendencies towards same-race non-best friendships began to weaken after a certain tipping point. Overall, differentiating between strong and weak tie homophily on various attributes and behaviors is apt to provide more insight into how broader social processes both shape and are shaped by adolescents’ friendship networks.

## Supplementary Material

1

## Figures and Tables

**Table 1.** Descriptive statistics at the individual-, network-, and school-cohort level

	Mean	SD	Min.	Max.
*Individual level (n = 25,590)*				
% Black	3.22%			
% Hispanic/Latino	6.49%			
% other race	5.70%			
% girl	51.34%			
% free/reduced lunch	27.63%			
% 6th grade	31.28%			
% 8th grade	38.16%			
% 11th grade	30.56%			
*Network level (n = 153)*				
Number of students	167.25	78.71	60	420
% racial minority	12.72	8.48	1.19	45.04
% female	51.13	4.41	34.52	63.16
*School-Cohort level (n = 51)*				
Proportion Rural	0.27	0.20	0.03	0.79
Money Spent per Student	8723.16	1795.54	6670	13237
% Pennsylvania	47.06%			
% Iowa	52.94%			
% Treatment	45.10%			
% Control	54.90%			

**Table 2.** Valued ERGM meta-analyses results

	Model 1		Model 2		Model 3	
	*b*	S.E.	*b*	S.E.	*b*	S.E.

*Dyad-level terms*						

Race						

General Homophily	0.128	(0.024)***			0.125	(0.027)***

Strong Tie Homophily			0.012	(0.049)		

Weak Tie Homophily			0.219	(0.032)***		

Gender						

General Homophily	1.322	(0.036)***	1.316	(0.033)***		

Strong Tie Homophily					0.963	(0.062)***

Weak Tie Homophily					1.400	(0.042)***

SES Homophily	0.164	(0.014)***	0.174	(0.015)***	0.170	(0.016)***

*Structural terms*						

Nonzero	−5.650	(0.072)***	−5.932	(0.064)***	−6.035	(0.069)***

Sum	−1.057	(0.020)***	−0.879	(0.049)***	−0.757	(0.037)***

Mutual	2.566	(0.037)***	2.561	(0.038)***	2.569	(0.039)***

Transitivity	0.847	(0.016)***	0.840	(0.016)***	0.861	(0.018)***

*n*	153		153		151	

Notes:

*** *p* <0.001.

Robust standard errors are reported.

**Table 3.** Independent variables of interest from MLMs for racial and gender homophily valued ERGM coefficients

	Models 1: General		Models 2: Weak Tie		Models 3: Strong Tie	
	*b*	S.E.	*b*	S.E.	*b*	S.E.

*Racial homophily models*						

% Racial minority	−0.024	(0.023)	−0.100	(0.032)**	0.025	(0.033)†

% Racial minority squared	0.002	(0.001)	0.005	(0.002)**	−0.001	(0.002)†

% Racial minority cubed	−0.00003	(0.00002)	−0.00006	(0.00003)*	0.00002	(0.00003)†

*n*	153		152		152	

*Gender homophily models*						

% Female	0.034	(0.092)	0.131	(0.106)	0.232	(0.140)

% Female squared	−0.0004	(0.0009)	−0.0014	(0.0010)	−0.0024	(0.0014)

*n*	153		150		150	

Notes:

* *p* <0.05

** *p* <0.01.

† denotes that the coefficient is statistically significantly different than the corresponding coefficient in Model 2 according to a Chi-squared test (*p* < 0.05). Robust standard errors are reported. All MLMs include controls for grade, number of students, other homophily variants, school SES, proportion rural, state, and treatment status (see Supplemental Materials, Part A).

## Footnotes

1 While I use the term friendship to refer to respondents’ directed nomination patterns, it is important to note that friendship represents an inherently symmetrical relationship (Kitts & Leal, 2021). Alternatively, the directed nominations studied here can be interpreted as individuals’ sentiments or proxies for “aspirational” friendships (Ball & Newman, 2013).

2 Results were substantively similar when the quadratic and cubic polynomial terms were excluded (see Supplemental Materials, Part B).

3 Results were substantively similar when the quadratic polynomial was excluded (see Supplemental Materials, Part B).

4 Roughly 11% of respondents nominated the maximum number of best and other friends in the current sample, and as a result, may not have been able to list all their friends on the survey.
