# Supplementary material for: Strong and weak tie homophily in adolescent friendship networks: An analysis of same-race and same-gender ties
Source: Netw Sci (Camb Univ Press). Author manuscript; Available in PMC 2023 Apr 14. (PMC10104515; doi:10.1017/nws.2022.24)
Supplement: 1 [file NIHMS1829975-supplement-1.pdf]

Strong and Weak Tie Homophily in Adolescent Friendship Networks: An Analysis of Same-Race and Same-Gender Ties

Supplemental Materials

Contents:

Part A. MLM results (full tables)

Part B. Robustness checks

Part C. Valued ERGM Goodness of Fit

Part A. MLM results (full tables)

Table S1. MLMs for racial homophily coefficients.

	**Model 1 General Homophily**			**Model 2 Weak Tie Homophily**			**Model 3 Strong Tie Homophily**		
	b	S.E.		*b*	S.E.		*b*	S.E.	
% Racial minority	-0.024	(0.023)		-0.100	(0.032)	**	0.025	(0.033)	†
% Racial minority squared	0.002	(0.001)		0.005	(0.002)	**	-0.001	(0.002)	†
% Racial minority cubed	-0.00003	(0.00002)		-0.00006	(0.00003)	*	0.00002	(0.00003)	†
Eighth grade	-0.008	(0.053)		-0.100	(0.074)		-0.109	(0.084)	
Eleventh grade	0.088	(0.058)		0.052	(0.080)		-0.109	(0.091)	
Number of students (100s)	-0.024	(0.036)		-0.019	(0.050)		0.075	(0.052)	
Gender homophily	-0.036	(0.056)		-0.120	(0.077)		-0.150	(0.086)	
SES homophily	-0.056	(0.103)		0.100	(0.142)		0.208	(0.158)	
% Rural	-0.287	(0.124)	*	-0.582	(0.172)	*	-0.016	(0.171)	†
School SES (in $1,000)	-0.006	(0.015)		0.005	(0.021)		-0.001	(0.021)	
Pennsylvania	-0.009	(0.056)		-0.117	(0.077)		-0.161	(0.078)	*
Treatment	0.062	(0.049)		0.119	(0.068)		0.005	(0.068)	
Intercept	0.393	(0.228)		1.057	(0.317)	**	0.092	(0.330)	
District-Level Intercept	0.010	(0.006)		0.019	(0.012)		0.008	(0.013)	
n	153			152			152		

Notes: **p* < 0.05, ***p* < 0.01, ****p* < 0.001. † denotes that the coefficient in Model 3 is statistically significantly different that the corresponding coefficient in Model 2 according to a Chi squared test (*p* < 0.05). Robust standard errors are reported. Omitted grade category is sixth grade.

Table S2. MLMs for gender homophily coefficients.

	Model 1 General Homophily			Model 2 Weak Tie Homophily			Model 3 Strong Tie Homophily		
	b	S.E.		*b*	S.E.		*b*	S.E.	
% Female	0.034	(0.092)		0.131	(0.106)		0.232	(0.140)	
% Female squared	-0.0004	(0.0009)		-0.0014	(0.0010)		-0.0024	(0.0014)	
Eighth grade	-0.478	(0.074)	***	-0.437	(0.081)	***	-0.480	(0.108)	***
Eleventh grade	-0.651	(0.073)	***	-0.600	(0.081)	***	-0.869	(0.107)	***†
Number of students (100s)	-0.001	(0.041)		-0.052	(0.046)		-0.225	(0.060)	***†
Racial homophily	0.052	(0.122)		0.114	(0.134)		0.356	(0.177)	*
SES homophily	-0.127	(0.151)		-0.035	(0.166)		0.188	(0.220)	
% Rural	-0.051	(0.166)		0.016	(0.182)		0.117	(0.241)	
School SES (in $1,000)	-0.005	(0.019)		-0.011	(0.021)		0.019	(0.028)	
Pennsylvania	-0.258	(0.067)	***	-0.257	(0.074)	***	-0.185	(0.098)	
Treatment	0.029	(0.060)		0.079	(0.067)		-0.081	(0.088)	†
Intercept	1.461	(2.307)		-0.894	(2.670)		-3.666	(3.529)	
District-Level Intercept	0.001	(0.011)		0.001	(0.026)		0.001	(0.011)	
n	153			150			150		

Notes: *$p < 0.05$, **$p < 0.01$, ***$p < 0.001$. † denotes that the coefficient in Model 3 is statistically significantly different that the corresponding coefficient in Model 2 according to a Chi squared test ($p < 0.05$). Robust standard errors are reported. Omitted grade category is sixth grade.

Part B. Robustness checks

Table S3. Supplemental valued ERGM meta-analyses that consider only 2 of the other non-best friendship nominations as weak ties for a subsample of networks.

	Model 2			Model 3		
	b	S.E.		*b*	S.E.	
Dyad-Level Terms						
Race						
General Homophily				0.148	(0.037)	***
Strong Tie Homophily	0.070	(0.039)				
Weak Tie Homophily	0.221	(0.043)	***			
Gender						
General Homophily	2.105	(0.057)	***			
Strong Tie Homophily				1.970	(0.074)	***
Weak Tie Homophily				2.237	(0.062)	***
SES Homophily	0.197	(0.027)	***	0.183	(0.027)	***
Structural Terms						
Nonzero	-7.878	(0.120)	***	-7.977	(0.157)	***
Sum	0.061	(0.041)		0.269	(0.071)	***
Mutual	2.249	(0.041)	***	2.272	(0.045)	***
Transitivity	0.642	(0.022)	***	0.640	(0.028)	***
n	50			47		

Notes: ***$p < 0.001$. Robust standard errors are reported. Models only include networks collected during students' 6th grade years.

Table S4. Valued ERGM meta-analysis results with interactions to test for variations in general, strong, and weak tie homophily by grade level

	Model 1			Model 2			Model 3		
	b	S.E.		*b*	S.E.		*b*	S.E.	
Race									
General homophily (baseline)	0.101	0.033	**				0.115	0.027	***
General homophily × 8th grade	0.019	0.040					-0.018	0.033	
General homophily × 11th grade	0.084	0.045					0.065	0.049	
Strong tie homophily (baseline)				0.026	0.070				
Strong tie homophily × 8th grade				-0.042	0.085				
Strong tie homophily × 11th grade				0.006	0.079				
Weak Tie Homophily (baseline)				0.191	0.029	***			
Weak tie homophily × 8th grade				0.009	0.033				
Weak tie homophily × 11th grade				0.102	0.048	*			
Gender									
General homophily (baseline)	1.738	0.043	***	1.690	0.055	***			
General homophily × 8th grade	-0.419	0.057	***	-0.397	0.075	***			
General homophily × 11th grade	-0.670	0.065	***	-0.606	0.064	***			
Strong tie homophily (baseline)							1.455	0.101	***
Strong tie homophily × 8th grade							-0.507	0.126	***
Strong tie homophily × 11th grade							-0.715	0.121	***
Weak Tie Homophily (baseline)							1.787	0.050	***
Weak tie homophily × 8th grade							-0.420	0.068	***
Weak tie homophily × 11th grade							-0.596	0.078	***

Notes: **p* < 0.05, ***p* < 0.01, ****p* < 0.001. Robust standard errors are reported. All valued ERGMs include controls for SES homophily, as well as the nonzero, sum, mutual, and transitivity network terms.

Table S5. MLM for racial homophily coefficients with no higher order polynomials for percent racial minority.

	Model 1 General Homophily			Model 2 Weak Tie Homophily			Model 3 Strong Tie Homophily		
	b	S.E.		*b*	S.E.		*b*	S.E.	
% Racial minority	0.007	(0.003)	*	0.010	(0.005)	*	0.014	(0.005)	**
Eighth grade	-0.005	(0.053)		-0.105	(0.076)		-0.112	(0.084)	
Eleventh grade	0.084	(0.051)		0.038	(0.083)		-0.107	(0.090)	
Number of students (100s)	-0.021	(0.037)		-0.005	(0.055)		0.074	(0.051)	
Gender homophily	-0.033	(0.101)		-0.135	(0.080)		-0.161	(0.085)	
SES homophily	-0.085	(0.081)		0.155	(0.147)		0.191	(0.155)	
% Rural	-0.284	(0.126)	*	-0.545	(0.190)	**	-0.011	(0.170)	
School SES (in $1,000)	-0.004	(0.015)		0.015	(0.023)		-0.001	(0.020)	
Pennsylvania	-0.015	(0.056)		-0.088	(0.084)		-0.146	(0.076)	
Treatment	0.049	(0.048)		-0.030	(0.073)		-0.000	(0.065)	
Intercept	0.238	(0.194)		0.408	(0.291)		0.129	(0.277)	
District-Level Intercept	0.011	(0.007)		0.029	(0.014)		0.008	(0.013)	
n	153			152			152		

Notes: **p* < 0.05, ***p* < 0.01, ****p* < 0.001. Robust standard errors are reported. Omitted grade category is sixth grade.

Table S6. MLM for racial homophily coefficients with second-order polynomial for percent racial minority.

	Model 1 General Homophily			Model 2 Weak Tie Homophily			Model 3 Strong Tie Homophily		
	b	S.E.		*b*	S.E.		*b*	S.E.	
% Racial minority	0.006	(0.009)		-0.034	(0.013)	*	0.005	(0.011)	
% Racial minority squared	0.000	(0.000)		0.001	(0.000)	***	0.000	(0.000)	
Eighth grade	-0.005	(0.047)		-0.093	(0.086)		-0.111	(0.084)	
Eleventh grade	0.084	(0.058)		0.045	(0.080)		-0.106	(0.090)	
Number of students (100s)	-0.022	(0.037)		-0.016	(0.052)		0.072	(0.051)	
Gender homophily	-0.029	(0.056)		-0.101	(0.078)		-0.155	(0.085)	
SES homophily	-0.033	(0.102)		0.146	(0.142)		0.190	(0.156)	
% Rural	-0.285	(0.127)	*	-0.578	(0.179)	**	-0.018	(0.171)	
School SES (in $1,000)	-0.005	(0.015)		0.010	(0.019)		-0.002	(0.021)	
Pennsylvania	-0.016	(0.057)		-0.133	(0.080)		-0.155	(0.077)	*
Treatment	0.051	(0.050)		0.093	(0.070)		0.012	(0.067)	
Intercept	0.248	(0.205)		0.717	(0.288)	*	0.191	(0.292)	
District-Level Intercept	0.012	(0.007)		0.023	(0.013)		0.008	(0.013)	
n	153			152			152		

Notes: **p* < 0.05, ***p* < 0.01, ****p* < 0.001. Robust standard errors are reported. Omitted grade category is sixth grade.

Table S7. MLM for gender homophily coefficients with no higher order polynomials for percent female.

	Model 1 General Homophily			Model 2 Weak Tie Homophily			Model 3 Strong Tie Homophily		
	b	S.E.		*b*	S.E.		*b*	S.E.	
% Female students	-0.010	(0.007)		-0.010	(0.008)		-0.009	(0.010)	
Eighth grade	-0.478	(0.074)	***	-0.440	(0.082)	***	-0.485	(0.108)	***
Eleventh grade	-0.649	(0.073)	***	-0.595	(0.081)	***	-0.860	(0.108)	***
Number of students (100s)	0.003	(0.041)		-0.041	(0.045)		-0.206	(0.060)	**
Racial homophily	-0.055	(0.121)		0.108	(0.134)		0.346	(0.178)	
SES homophily	-0.127	(0.151)		-0.036	(0.167)		0.185	(0.222)	
% Rural	-0.065	(0.163)		-0.021	(0.181)		0.055	(0.240)	
School SES (in $1,000)	-0.005	(0.019)		-0.010	(0.021)		0.021	(0.028)	
Pennsylvania	-0.257	(0.067)	***	-0.257	(0.074)	**	-0.186	(0.098)	
Treatment	-0.032	(0.060)		0.088	(0.066)		-0.065	(0.088)	
Intercept	2.549	(0.373)	***	2.651	(0.426)	***	2.359	(0.566)	***
District-Level Intercept	0.003	(0.011)		0.011	(0.025)		0.004	(0.011)	
n	153			150			150		

Notes: **p* < 0.05, ***p* < 0.01, ****p* < 0.001. Robust standard errors are reported. Omitted grade category is sixth grade.

Part C. Valued ERGM Goodness of Fit

I present diagnostics from a representative friendship network for each of the three sets of ERGMs discussed in the manuscript. For each model, I include a correlation matrix for all variables, as well as each parameter's respective VIF (see Tables S4, S5, and S6). In general, diagnostics are favorable. While some parameters are highly correlated, VIFs suggest that the ERGMs are not at high risk of multicollinearity. The majority of VIFs are less than 20, suggesting that there is a low risk of multicollinearity. Those VIFs that are greater than 20 are still below 150. This finding is reassuring since VIFs greater than 150 are understood to be highly concerning (Duxbury, 2018).

Reference:

Duxbury, S.W. (2018). Diagnosing multicollinearity in exponential random graph models." *Sociological Methods & Research*, doi: 10.1177/0049124118782543.

Table S7. Correlation matrix and VIFs for a valued ERGM with traditional homophily parameters estimated on a representative friendship network.

	**Correlations**							**VIFs**
	Nonzero	Sum	Same Race	Same Gender	Same SES	Recip.	Trans.	
Nonzero	1.00							
Sum	0.98	1.00						2.69
Same Race	0.98	0.96	1.00					1.14
Same Gender	0.99	0.98	0.97	1.00				1.09
Same SES	0.93	0.93	0.93	0.92	1.00			1.15
Reciprocity	0.91	0.95	0.90	0.91	0.88	1.00		2.67
Transitivity	0.94	0.97	0.94	0.94	0.91	0.96	1.00	2.64

Table S8. Correlation matrix and VIFs for a valued ERGM with strong and weak tie racial homophily parameters estimated on a representative friendship network.

	**Correlations**								**VIFs**
	Nonzero	Sum	Same Race High	Same Race Low	Same Gender	Same SES	Recip.	Trans.	
Nonzero	1.00								
Sum	0.98	1.00							14.94
Same Race High	0.83	0.92	1.00						6.38
Same Race Low	0.72	0.56	0.29	1.00					7.35
Same Gender	0.99	0.97	0.83	0.71	1.00				1.08
Same SES	0.89	0.86	0.74	0.70	0.88	1.00			1.12
Reciprocity	0.90	0.95	0.92	0.45	0.91	0.78	1.00		2.83
Transitivity	0.93	0.97	0.92	0.51	0.93	0.82	0.96	1.00	2.83

Table S9. Correlation matrix and VIFs for a valued ERGM with strong and weak tie gender homophily parameters estimated on a representative friendship network.

	**Correlations**								**VIFs**
	Nonzero	Sum	Same Race	Same Gender High	Same Gender Low	Same SES	Recip.	Trans.	
Nonzero	1.00								
Sum	0.98	1.00							40.76
Same Race	0.97	0.95	1.00						1.04
Same Gender High	0.84	0.93	0.82	1.00					27.50
Same Gender Low	0.84	0.71	0.82	0.42	1.00				18.32
Same SES	0.92	0.90	0.91	0.78	0.77	1.00			1.03
Reciprocity	0.91	0.95	0.89	0.92	0.61	0.84	1.00		2.27
Transitivity	0.93	0.96	0.92	0.91	0.67	0.87	0.94	1.00	2.19
